# NS3 genomic sequencing and phylogenetic analysis as alternative to a commercially available assay to reliably determine hepatitis C virus subtypes 1a and 1b

**DOI:** 10.1371/journal.pone.0182193

**Published:** 2017-07-28

**Authors:** Karin Neukam, Alfredo P. Martínez, Andrés C. A. Culasso, Ezequiel Ridruejo, Gabriel García, Federico A. Di Lello

**Affiliations:** 1 Universidad de Buenos Aires, Facultad de Farmacia y Bioquímica, Cátedra de Virología, Buenos Aires, Argentina; 2 Instituto de Biomedicina de Sevilla / Hospital Universitario Virgen del Rocío / CSIC / Universidad de Sevilla, Unit of Infectious Diseases, Microbiology and Preventive Medicine, Seville, Spain; 3 Hospital Universitario de Valme, Unit of Infectious Diseases and Mirobiology, Seville, Spain; 4 Centro de Educación Médica e Investigaciones Clínicas Norberto Quirno "CEMIC", Virology Section, Buenos Aires, Argentina; 5 Consejo Nacional de Investigaciones Científicas y Técnicas (CONICET), Buenos Aires, Argentina; 6 Centro de Educación Médica e Investigaciones Clínicas Norberto Quirno "CEMIC", Hepatology Section, Department of Medicine, Buenos Aires, Argentina; Chiba University, Graduate School of Medicine, JAPAN

## Abstract

**Objective:**

To evaluate the use of hepatitis C virus (HCV) NS3 sequencing as alternative to the comercially available Versant HCV 2.0 reverse hybridization line-probe assay (LiPA 2.0) to determine HCV genotype 1 (HCV-1) subtypes.

**Patients and methods:**

A cohort of 104 patients infected by HCV-1 according to LiPA 2.0 was analyzed in a cross-sectional study conducted in patients seen from January 2012 to June 2016 at an outpatient clinic in Buenos Aires, Argentina.

**Results:**

The samples were included within well supported subtype clades: 64 with HCV-1b and 39 with HCV-1a infection. Twenty of the HCV-1a infected patientes were included in a supported sub-clade “1” and 19 individuals were among the basal sub-clade “2”. LiPA 2.0 failed to subtype HCV-1 in 20 (19.2%) individuals. Subtype classification determined by NS3 direct sequencing showed that 2/18 (11.1%) of the HCV-1a-infected patients as determined by LiPA 2.0 were in fact infected by HCV-1b. Of the HCV-1b-infected according to LiPA 2.0, 10/66 (15.2%) patients showed HCV-1a infection according to NS3 sequencing. Overall misclassification was 14.3% (κ-index for the concordance with NS3 sequencing = 0.635). One (1%) patient was erroneously genotyped as HCV-1 and was revealed as HCV genotype 4 infection.

**Conclusions:**

Genomic sequencing of the HCV NS3 region represents an adequate alternative since it provides reliable genetic information. It even distinguishes between HCV-1a clades related to resistance-associated substitutions to HCV protease inhibitors, it provides reliable genetic information for genotyping/subgenotyping and simultaneously allows to determine the presence of resistance-associated substitutions to currently recommended DAAs.

## Introduction

In the era of direct-acting antivirals (DAA) against chronic hepatitis C virus (HCV) infection is important to reliably determine HCV genotype 1 (HCV-1) subtype since misclassification may result in an inadequate therapy [1–3]. The Versant HCV 2.0 reverse hybridization line-probe assay (LiPA 2.0) represents a widely used commercially available assay to determine HCV genotype. However, a number of studies have detected an important proportion of discrepancies in HCV-1 subtype determination when compared to genomic sequencing methods including HCV core, E1 and NS5B regions [4–9]. To date, there is no data on comparisons with the NS3 region, which could simultaneously provide information on resistance-associated substitutions (RASs).

The aim of this study was to evaluate NS3 sequencing in combination with phylogenetic analyses as alternative of LiPA 2.0 to determine HCV subtypes 1a and 1b in the setting of a Southern American population.

## Patients and methods

### Study population

From January/2012 to June/2016, 211 patients living in Buenos Aires City with positive HCV antibodies were seen at the Centro de Educación Médica e Investigaciones Clínicas Norberto Quirno "CEMIC" in Argentina. In this retrospective cross-sectional study, those who fulfilled the following criteria were included: i) older than 18 years; ii) infected with HCV-1 as determined by LiPA 2.0 and iii) plasma sample frozen at -80°C available.

### HCV genotype determination by LiPA 2.0

Plasma HCV-RNA was extracted with the COBAS^®^ AmpliPrep Total Nucleic Acid Isolation Kit (Cobas TaqMan; Roche Diagnostic Systems Inc., Pleasanton, CA, USA). The extracted RNA was then amplified with the Versant HCV LiPA 2.0 Amplification Kit (Siemens, Tarrytown, NY, USA) in order to perform the reverse transcription (RT) PCR hybridization assay. HCV genotype was determined by the Auto-LiPA 48 Instrument and Autoblot 3000H tests (Siemens, Tarrytown, NY, USA). All procedures were carried out according to the manufacturer’s instructions.

### RT-PCR and sequencing

RNA was extracted using the MagNA Pure system (Roche Diagnostics, Mannheim, Germany). The partial NS3 region was amplified by heminested PCRs using MMLV-RT (Promega, Madison, WI, USA) with Random Hexamer Primers. The first PCR was performed with 5μl of cDNA using primers ES3426 and EA4140 (Table 1). For the second PCR, 3μl of the first PCR product and primers ES3426 and IA4011 (Table 1) were used. The PCR conditions for the first round amplification were: 30 cycles at 95°C for 45s, 50°C for 45s, and 72°C for 60s. The PCR conditions for the second round amplification were: 30 cycles at 95°C for 45s, 55°C for 45s, and 72°C for 45s. Denaturation at 95°C for 5 min and a final extension at 72°C for 10 min were performed in both rounds of amplification. Amplified DNAs were subsequently purified from agarose gels by the QIAquik Gel Extraction Kit and then sequenced in both senses by INTA (Castelar, Argentina).

**Table 1:** Primers used for amplification of partial NS3 region of the hepatitis C virus genome.

Primers designation	Sequence (5´→3´)	Genome position*
Outer primers		
ES3426	ATC ACG GCS TAY KCC CAR CAG AC	3426–48
EA4140	CCA TGK GCC TTR GAC ATR TA	4120–40
Inner primers		
ES3426	ATC ACG GCS TAY KCC CAR CAG AC	3426–48
IA4011	CAA GTG GCC CAT CTA CAC GC	3992–4011

*Nucleotide position according to reference sequence H77 (GenBank accession number AF009606).

### Phylogenetic analysis

The HCV sequences were phylogenetically analyzed to determine its genotype. First, a reference dataset, composed by 174 well characterized full length genome sequences, was obtained from the NCBI Viral Genotyping Tool (https://www.ncbi.nlm.nih.gov/projects/genotyping/formpage.cgi). Then, the dataset was reduced to 48 full length genomes. The criteria for inclusion in the reduced dataset was the following: 1) The genome was classified as subtype “a”, “b” or “c”; 2) for those subtypes with many sequences, only 6 were randomly picked; 3) for subtype 1a, 10 sequences were used, five of each subclade described by Pickett and colleagues [10]. Second, all sequences (reference and samples) were aligned with Muscle 3.8.31 [11]. The resulting alignment was trimmed to the length of the NS3 region analyzed in this work. Third, a model selection procedure was carried out with jModelTest v 2.1.10 [12]. Finally, the best fit model according with the “Bayesian Information Criterion” (GTR+Γ4) was used to perform the actual phylogenetic reconstruction by Maximum Likelihood methodology implemented in PhyML v 3.0.1 [13]. The branch support value were assessed by bootstrapping (1000 pseudoreplics).

### Statistics

The κ-index for the concordance of subtype determination between NS3 sequencing and LiPA 2.0 was calculated. A κ-index of 1 indicates perfect agreement; 1 ≥ k > 0.75 almost perfect; 0.75 ≥ k >0.45 substantial; 0.45 ≥ k > 0.2 moderate; 0.2 ≥ k >0 fair and k = 0 indicates no agreement [14]. The specificity, sensibility, positive predictive value (PPV) and negative predictive value (NPV) for the determination of HCV-1 subtype was calculated in those patients with a subtype determination obtained by LiPA 2.0. Statistical analysis was performed using the SPSS statistical software package release 23.0 (IBM, Chicago, IL, USA) and Fisterra.com (Elsevier 2012; http://www.fisterra.com/mbe/investiga/pruebas_diagnosticas/pruebas_diagnosticas.asp).

### Ethical aspects

Written informed consents to participate in this study were obtained from the patients. The study protocol was approved by the ethics committee of the “Facultad de Farmacia y Bioquímica de la Universidad de Buenos Aires” (record number 732575/2010) in accordance with the Helsinki Declaration.

## Results

### Study population

A total of 104 patients were included in this study. Median (Q1-Q3) age was 53.4 (46–61.7) years, 63 (60.6%) were male and no patient showed HIV coinfection. Median (Q1-Q3) baseline HCV-RNA was 5.9 (5.4–6.5) log_10_ IU/mL.

### Phylogenetic analysis

**Fig 1:**
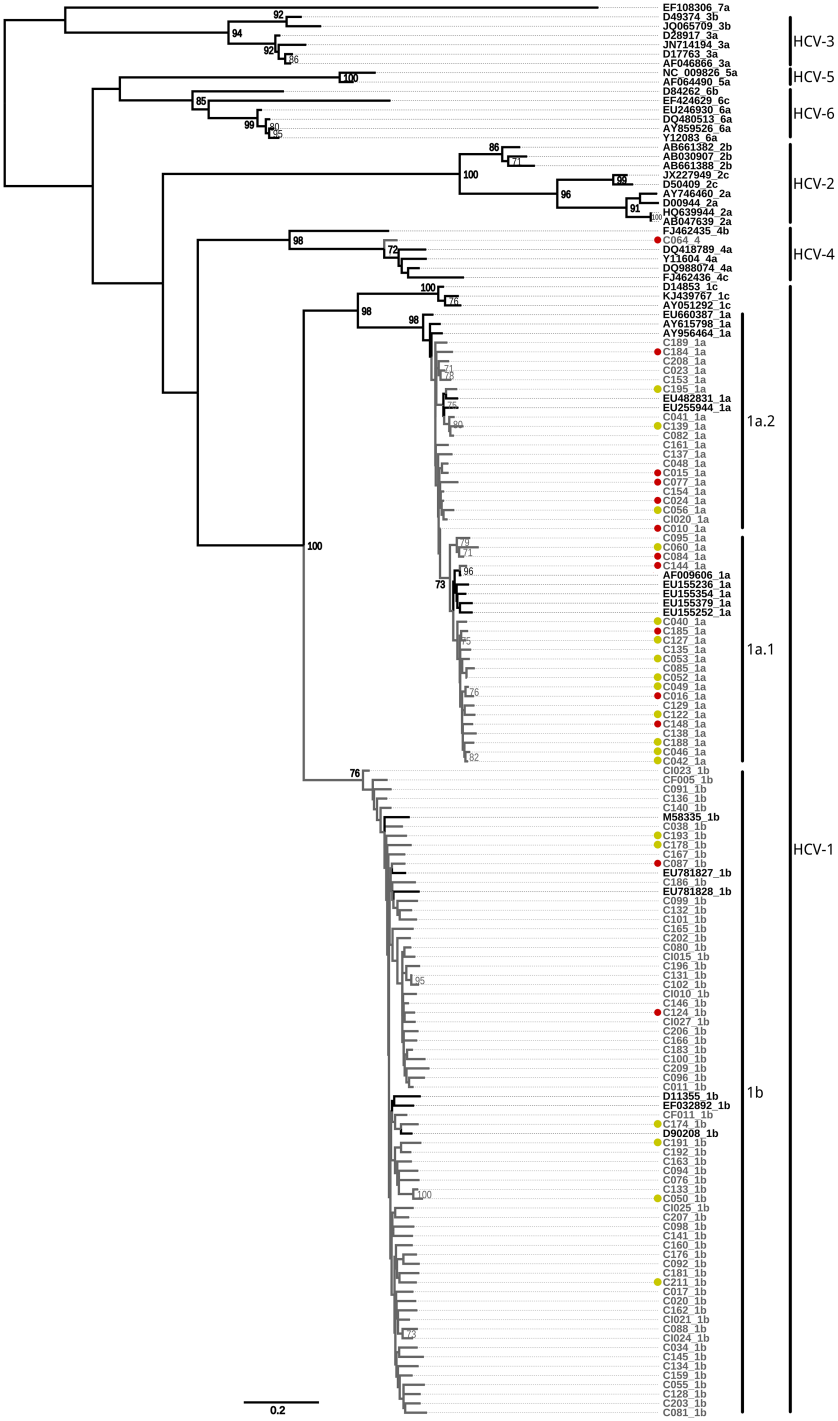
Maximum likelihood phylogenetic tree (GTR+Γ+I model for nucleotide substitutions) including 104 the new HCV-NS3 samples of this study (in gray) and 48 references sequences (in black) from the NCBI Viral Genotyping Tool (https://www.ncbi.nlm.nih.gov/projects/genotyping/formpage.cgi). The branch lengths are proportional to the evolutionary distance between the samples. The numbers at each node correspond to bootstrap values obtained with 1,000 replicates (values lower than 70 are not shown). Yellow dots indicate samples that remained undetermined by LiPA 2.0 while red dots indicate samples that were misclassified by LiPA 2.0.

The phylogenetic tree of NS3 showed supported clusters (bootstrap >70) for each genotype and subtype, excepting genotype 4, of which subtypes were not supported. The majority samples were included within well supported subtype clades: 64 with HCV-1b and 39 with HCV-1a infection. The remaining sample was genotypified as “4”. Twenty out of the 39 HCV-1a infected patientes were included in a supported sub-clade “1” of HCV-1a while the remaining 19 individuals were among the basal sub-clade “2” (Fig 1). Additional analyses carried out by neighbor joining and distance methods resulted in trees were both HCV-1a clades were sister. However, the ML methology proved to outperform NJ and thus we prefer this topology where clade 2 is basal to clade 1. In either case the clade 1 sequences are differentiable from clade 2.

### Nucleotide sequences accession numbers

Nucleotide sequences for the HCV NS3 have been deposited in GenBank under accession numbers KY614603—KY614705.

### Concordance of sequencing assays and phylogenetic analysis with LiPA 2.0

According to LiPA 2.0, 18 (17.3%) individuals were infected by HCV-1a and 66 (63.5%) patients by HCV-1b. HCV-1 subtype remained undetermined in 20 (19.2%) individuals. Subtype classification determined by NS3 direct sequencing showed that 11.1% of the HCV-1a-infected patients as determined by LiPA 2.0 were in fact infected by HCV-1b. Of those individuals classified as infected by HCV-1b according to LiPA 2.0, 15.2% patients were misclassified and showed HCV-1a infection according to NS3 sequencing. Numbers of concordant and discordant results are summed up in Table 2.

**Table 2:** Agreement and discordance of HCV genotyping by means of NS3 direct sequencing and LiPA 2.0.

HCV genotype according to LiPA 2.0	HCV genotype according to NS3 direct sequencing			Total
	1a	1b	4	
1a	16	2	0	18
1b	10	56	0	66
Subtype not determined	13	6	1	20
Total	39	64	1	104

In the group of the 84 patients in whom a HCV-1 subtype determination could be obtained by LiPA 2.0, the κ-index for the concordance of subtype determination with NS3 sequencing was 0.635, the overall misclassification rate was 14.3%. The predictive values for HCV-1a subtyping by LiPA 2.0 were as followed: specificity: 96.6% (95%CI: 87.1%-99.4%); sensibility: 61.5% (95%CI: 40.7%-79.1%); PPV: 88.9% (95%CI: 63.9%-98.1%) and NPV: 84.9% (95%CI: 73.4%-92.1%). Corresponding figures for HCV-1b subtyping were: specificity: 61.5% (95%CI: 40.7%-79.1%); sensibility: 96.6% (95%CI: 87.1%-99.4%); PPV: 84.9% (95%CI: 73.4%-92.1%) and NPV: 88.9% (95%CI: 63.9%-98.1%), respectively.

Among the patients in whom HCV-1 subtype was undetermined, 6 (5.8%) individuals were infected by HCV-1a and 13 (12.5%) were infected by HCV-1b as determined by NS3 sequencing. One (1%) patient was erroneously genotyped as HCV-1 and was revealed as HCV genotype 4 infection by sequencing (Fig 1).

## Discussion

The data presented herein show that NS3 sequencing represents an adequate and more accurate method that LiPA to determine HCV subtypes 1a and 1b, which is crucial for treatment decision. The phylogenetic analysis of this region also allowed distinguishing between the two clades of HCV-1a which present differences in RAS frequencies. This technique furthermore allows to simultaneously detect RASs, thus having a high clinical value.

The utilization of DAA against chronic HCV infection has led to very high overall sustained virologic response rates [15]. However, there is no unique regimen for all settings and the selection of drugs and decision of treatment duration partly depend on the HCV genotype and subtype. In the case of HCV-1 infection, guidelines clearly distinguish between the HCV-1a and HCV-1b subtypes [1–3]. Therefore, recently there is an evident interest in developing reliable methods [5–8,16–18]. The herein presented results show that NS3 sequencing does not only give good phylogenetic information but also it allows distinguishing between the two clades of HCV-1a. Additionally, the presence of RASs has become a major concern in DAA-based therapy, since it currently represents one of the main reasons for treatment failure. According to European Association for the Study of the Liver (EASL) guidelines, the presence of RASs affects three out of five widely used treatment regimens [1]. This is true both for treatment-naïve patients, as well as for treatment-experienced individuals. However, RASs are not equally distributed among the different HCV genotypes and subtypes and the determination of specific RASs for treatment decision is restricted to HCV-1a [1–3]. NS3 sequencing allows determining RASs within the same procedure. Finally, apart from the clinical benefit, the application of this sequencing method results in cost reduction of 50–70% as compared to LiPA. For all these reasons, genotyping by direct sequencing of NS3 should be considered in order to optimize treatment options and prognosis.

LiPA 2.0 targets both the 5′UTR and the HCV core regions in order to avoid misclassifications. However, the herein presented results suggest that LiPA 2.0 assay is not an accurate method for subtyping HCV-1. First, the assay failed to subgenotype one fifth of the studied population. Second, in a considerable proportion of individuals, HCV-1 subtypes were misclassified. Although the proportions of both HCV-1 misclassification, as well as indeterminate calls vary considerably between reports [4–9,16,19], the misclassification rate of 14% observed in this study is in accordance with a number of previously published studies [4,7–9]. Since the misclassification of HCV-1 subtype might lead to inadequate DAA choice or no testing of RASs, sequencing should be considered for HCV-1 infected patients. Importantly, in the clinical practice, incorrect genotyping can lead to DAA treatment failure [20] and/or exposure to a drug that is not efficient, resulting in decreased patient safety and economic disadvantages. In fact, an HCV genotype 4-infected patient was erroneously genotyped as HCV-1 by LiPA 2.0 herein and may have received a combination based on dasabuvir, a drug efficient in HCV-1 but not indicated for genotype 4 [1–3]. This finding is in agreement with that previously reported by Guelfo and colleagues, who also observed a patient erroneously genotyped by LiPA [4]. Therefore, although the concordance between NS3 sequencing and LiPA could mathematically be classified as “substancial”, it is not acceptable in the clinical practice. Something similar can be said about the predictive values for the easier-to-treat HCV subtype 1b, where a PPV of 85% was observed. Hence, relying on LiPA 2.0 results, an important proportion of HCV-1a infected patients may receive therapy not sufficiently efficient since tailored for HCV-1b infection, while a considerable proportion of HCV-1b-infected patients may be overtreated if erroneously classified as HCV-1a. Although pangenotypic drugs will be used to an increasing degree in the future, it has to be taken into account that the availability of these drugs is limited in some settings, especially low-income countries. Furthermore, the HCV 1 subtype indicates if RAS testing is recommended. Therefore, there is still need for genotyping at the present time.

The reason for failure or misclassification by LiPA can merely be speculated. Since the first commercial version of LiPA genotyping had several problems, including misclassification of HCV-1a and 1b, LiPA was modified by including probes from the core region, resulting in the LiPA 2.0. Still, the absence in the current version of the regions with the most adequate variability may lead to the failure observed. Indeed, Chantratita and colleagues observed that the HCV genotypes 1a, 1b, 2, 3, 4, and 6 along with their subtypes have more than 95% sequence homology. Thus, the high percentage of sequence homology is a likely reason for the discrepancies in the results of LiPA 2.0 assay [6]. Additionally, the misclassification of genotype 1a as 1b or vice versa has been described on the basis of the sequence polymorphism at position -99 of the genome, frequently used to differentiate genotypes 1 [21].

In conclusion, NS3 sequencing represents an adequate technique to determine the HCV-1 subtypes and simultaneously determine RASs. Given the influence of the HCV-1-subtype in the DAA treatment response and the necessity of testing for baseline RASs in specific settings of HCV-1 infection, this technique should be considered for its use in the clinical practice.
